# Ethnic differences in treatment outcome for children and young people accessing mental health support

**DOI:** 10.1007/s00787-023-02233-5

**Published:** 2023-05-28

**Authors:** H. Ruphrect-Smith, S. Davies, J. Jacob, J. Edbrooke-Childs

**Affiliations:** 1 Clinical, Educational, and Health Psychology, University College London, London, UK; 2 Evidence Based Practice Unit, University College London and the Anna Freud Centre, Anna Freud, 4-8 Rodney Street, London, N1 9JH UK; 3 Child Outcomes Research Consortium, Anna Freud, London, UK

**Keywords:** Children, Young people, Mental health, Ethnic, Minoritized, Measurable change

## Abstract

Children and Young People (CYP) from minoritized ethnic backgrounds experience structural inequalities in Children and Young People’s Mental Health Settings (CYPMHS). This mixed methods study explores whether CYP’s ethnicity is associated with their treatment outcomes (operationalised as ‘measurable change’) from CYPMHS. A multilevel multi-nominal regression analysis, controlling for age, gender, referral source, presenting difficulty, case closure reason, suggests that CYP from Asian backgrounds (OR = 0.82, CI [0.70, 0.96]) and Mixed-race (odds ratio (OR) = 0.80; 95% CI [0.69, 0.92]) are less likely to report measurable improvement in mental health difficulties compared to White British CYP. Three themes from a thematic analysis of semi-structured interviews with 15 CYP from minoritized ethnic backgrounds focused on views and experiences of ending mental health support are also presented. CYP view personalised support and the right therapist as conducive to good endings and valued a range of outcomes pertaining to empowerment. Experiences of stigma and inequalities may begin to explain the less positive outcomes experienced by Asian and Mixed-race CYP found in the regression analysis. The implications of these findings and future areas of research are suggested.

**Supplementary Information:**

The online version contains supplementary material available at 10.1007/s00787-023-02233-5.

## Introduction

It is a moral and legal imperative that mental health services do not discriminate between  individuals or groups of children and young people (e.g., Article 2, United Nations Convention on the Rights of the Child [1]). Equitable care is pivotal to high levels of healthcare quality for all [2] which is cited as a priority for CYP’s mental health services (CYPMHS) in the UK (i.e., individuals aged ≤ 25 years) [3]. However, incongruent with these care equality aspirations, there is limited international research focused on this important area, with most notable research located in the US [4–7]. Recent research in the UK suggests that CYP’s ethnic background is associated with differing experiences of CYPMHS [8, 9]. Research in the US also suggests that access to CYPMHS and treatment outcomes for CYP in Black, Hispanic and Asian American communities is due to acculturation, social stigma, ethnic disparities in diagnosis, and a lack of culturally sensitive treatments [4, 6]. Central barriers from the adult literature include a perceived inability of services to deliver culturally competent care [10, 11], mental health stigma [12–15] and associated fear [16], and discrimination or communication barriers [17, 18]. Notably, experiences of stigma varies between minoritized ethnic groups [19]. For example, in the UK, Asian American CYP and British Asian families, CYP have been found to be more likely to seek help but less likely to make this known for fear of damaging their family reputation or enduring parental disappointment [4] in accessing CYPMHS [12]. Further, research also suggests that better treatment outcomes are reported when culturally competent care is provided in line with gender, ethnicity and language [20]. Yet, further understanding is required to understand the nuances between ethnicity and differing treatment outcomes.

Linked to this, disparities between ethnic backgrounds for reasons for treatment ending have also been demonstrated. For example, CYP from minoritized ethnic backgrounds have been found to be 9.5 times more likely to disengage from treatment compared to White American CYP [7]. Further, CYP from minoritized ethnic backgrounds experience different referral routes and case closure reasons from CYPMHS compared to White British CYP [8, 21]. For example, one study including CYP with a range of presenting difficulties and controlling for socioeconomic deprivation found CYP from Black, Asian, and Mixed-race backgrounds were less likely to be referred through primary care than educational and social care or youth justice services, compared to White British CYP [8]. Further, in the US, African American CYP have been found to be significantly more likely than their White counterparts to enter CYPMHS via the children’s services sector [21]. A meta-analysis including 1,953,135 participants found individuals (≥ 13-years-old) from minoritized ethnic backgrounds were significantly more likely to experience compulsory hospital admission (under the Mental Health Act), than their White British counterparts [22].

Pertinently, primary care referrals to specialist services (e.g., CYPMHS), more likely received for White CYP, are typically *voluntary* (arising following concerns of an individual’s behaviour or emotional state). Conversely, other referral routes (e.g., educational, social care or youth justice services), more likely received for CYP from minoritized ethnic backgrounds, are typically *compulsory* (resulting from concerns about risk of harm to self or others) and crucially, may be associated with more enduring difficulties and poorer outcomes [8, 21]. Although the cause of differences in referral routes is unclear, they are likely a consequence of the structure of services and broader societal factors that mediate how individuals from different ethnic backgrounds have poorer treatment outcomes in CYPMHS [23]. Being forced into treatment via third party referral sources, removing choice, or notions of a safe therapeutic environment could in turn, impact on engagement and overall treatment outcomes. Additionally, not accounting for cultural differences in delivery, could reinforce structural inequalities instead of empowering CYP from minoritized ethnic backgrounds towards positive treatment outcomes [24]. The Health Disparities and Outcomes (HDO) model demonstrates that experiences of treatment outcome are in part determined by access experiences [25]. This is based on the Gateway Provider Model that demonstrates a patient’s referral route is one of several health-care encounters determining treatment outcomes [26]. Thus, the broader literature supports the suggestion that ethnic inequalities in referral routes may be indicative of inequalities in outcomes. As mental health difficulties in youth are associated with worse adult quality of life and lower net income [27–29], the existence of inequalities in treatment outcome are likely to contribute to ongoing ethnic inequalities in society [30]. Crucially, there is a gap in the extant literature on whether ethnicity is associated with differential treatment outcomes for CYP accessing CYPMHS. Exploring this is a first step in working towards the essential aim of overcoming ethnic inequalities in mental health services and in society.

An individual’s treatment outcome is commonly determined based on the difference between a baseline and discharge recording on a given outcome measure [31]. Historically, the effect size differences have been used to evidence progress or change [32]. Using effect sizes, however, does not account for individual-level differences in outcome, which could produce under or overestimates of treatment efficacy for individuals within a group [33]. A large meta-analysis assessing youth psychological therapies used effect sizes to report that CYP’s ethnicity had no association with treatment outcome, stating psychological therapies are equally efficacious across ethnicity, albeit recognising an underrepresentation of CYP from minoritized ethnic backgrounds in the research [34]. However, failing to operationalise treatment outcome with a metric that accounts for individual-level differences may have led to an underestimate of the differential efficacy of treatment for CYP from different ethnic backgrounds. Accordingly, there is a need to explore the ethnicity-outcome association using an operationalisation of treatment outcome accounting for individual-level differences [35]. ‘Reliable Change’ is an increasingly favoured metric for operationalising treatment outcome, because it accounts for individual-level differences and accommodates the use of multiple outcome scales [36]. Reliable change considers the difference in individual’s scores between two timepoints on an outcome scale, controlling for the change attributable to that scale’s expected measurement error, providing an individual-level, reliable operationalisation of treatment outcome [37, 38]. Reliable change is limited to standardised measures of treatment outcome (i.e., those that are predefined, scoring individuals on fixed scales usually with norms) [39]. Such measures do not necessarily reflect CYP’s desired outcomes [40]. This may be partially redressed via the incorporation of idiographic measures (those that include personalised areas of outcome), such as the Goal-Based Outcome tool (GBO) [40], into outcome operationalisations [41]. In addition to using reliable change for standardised measures, the metric of measurable change (an extension of reliable change), allows for the inclusion of idiographic measures using a threshold defined by clinical experts [42]. Thus, measurable change incorporates multiple treatment outcome scales, accounts for individual-level differences, and is hoped to be clinically meaningful. Therefore, this study will fill a gap in the extant literature by examining if ethnicity is associated with differential treatment outcomes, operationalised as measurable change, for CYP accessing CYPMHS.

Although for over 20 years, UK mental health services have been legally obliged to overcome ethnic inequalities (e.g., Race Relations (Amendment) Act [43]), contemporary identification of ethnic inequalities in experiences of CYPMHS suggests that this is insufficient to overcoming them [5, 44]. Accordingly, to understand these issues in depth, with a view to changing policy, discussions are needed to identify experiences of ending mental health support valued by CYP from a diverse range of ethnic backgrounds [10, 45]. However, the qualitative literature has seldom investigated whether CYP from different ethnic backgrounds have varied views and experiences of ending mental health support. As such differences may help explain the potential association between ethnicity and treatment outcomes. CYP have described environments that enable them to open up and change their perspectives about difficulties they have were exceptionally transformative and promotional of good outcomes [46–48]. In contrast, it is suggested that parents/carers favour treatment endings that promote improved academic functioning, and therapists view changes in youth self-identity or self-confidence as indicative of good outcomes [49]. Indeed, in line with Strupp and Hadley’s [50] tripartite model of outcomes, evidence of differences in stakeholder’s valued outcomes is frequently observed in the literature [50–52]. While all three groups (CYP, parent/carer and practitioner) value reduced symptoms, and improved coping and resilence to some extent [49], expressing CYP’s valued outcomes may result in mismatches between practitioner and patient, resulting in end goals that may contribute to poor outcomes for CYP. This is a process to be worked through collaboratively. Further, given the endemic nature of systemic racism, patients from minoritized ethnic backgrounds may be aware of the role of structural inequalities but not explicitly mention their influence on their experience [53]. Consequently, the influence of ethnic inequalities may be absent in some qualitative explorations while still influencing individuals experiences of mental health support. The HDO and Gateway provider models illustrates how treatment endings can be influenced by experiences of accessing and attending care [25, 26]. Although studies have begun to elucidate CYP’s valued outcomes and endings of mental health support, this literature has focused on breadth (e.g., [49]), not depth. Moreover, the extant literature has not, to our knowledge, investigated how these mental health support experiences differ across CYP from different ethnic backgrounds.

### Aim

The aim of this study is to fill the gaps in the literature pertaining to whether ethnicity is associated with differential treatment outcomes for CYP accessing CYPMHS. The research question is: “What is the relationship between ethnicity and treatment outcome for CYP accessing CYPMHS?”. Based on the literature, the hypothesis is “CYP from minoritized ethnic groups will have worse treatment outcomes than White British CYP”.

## Method

Ethical approval was granted by the UCL Department of Psychology Ethics Committee (CEHP/2020/582).

### Quantitative data

#### Participants

Data for the secondary analysis were collated from the recently merged Child Outcomes Research Consortium (CORC) and Children and Young People’s Improving Access to Psychological Therapies (CYP IAPT) (2011–2015) datasets. Subsets of the overall data set/s have been analysed to address other research questions, e.g., [54, 55]. However, the subsample derived to explore the current research question was unique to this study. Cases were included in the analysis if the CYP was 6–25 years old (within the age range that relevant measures are self-reported), the case was closed, and the case had complete ethnicity data. As seen in Table 1, this resulted in a final sample of 14,534 CYP (7977 female, 6577 male) from 75 services, with a mean age of 11.82 (SD = 3.12) and an age range of 6–25 years. Ethnicity is routinely recorded by services using the 2001 census categories but was organised into superordinate groups for analysis as in Table 1. Data on socioeconomic status were not available in the dataset.

**Table 1:** Descriptive statistics for all quantitative variables

Demographic characteristics	*n*	%	
**Age** (M=11.82, SD=3.12, range=6-25)^a^	14,534	100	
**Gender**			
Female	7977	54.89	
Male	6557	45.11	
**Ethnicity**			
Asian	880	6.05	
Black	1468	10.1	
Mixed-race	1054	7.25	
Other	532	3.66	
White British	9826	67.61	
White other	774	5.33	

Clinical characteristics	*n*	%		*n*	%
**Referral source**^b^			**Presenting difficulties**		
Primary care	2638	18.15	Child in Need	392	2.70
Self-referral	2145	14.76	Child Protection Plan	802	5.52
Education	1160	7.98	Autism	399	2.75
Social care/youth justice	312	2.15	Conduct problems	1310	9.01
Child health	304	2.09	Developmental difficulties	1210	8.33
Mental health	872	6	Eating disorder	939	6.46
Other	264	1.82	Emotional problems	4499	30.96
Not reported	6839	47.06	Repetitive behaviour problems	1401	9.64
**Case closure reason**^c^			Hyperactivity	1024	7.05
Mutual agreement	3627	24.96	Learning difficulties	326	2.24
Nonattendance	616	4.24	Psychosis	650	4.47
Onward referral	345	2.37	Self-harm	1902	13.09
Other	471	3.24	Substance use	298	2.05
Not reported or not known	9475	65.19	Other problems	3125	21.05
**Reliable change**					
No change	7468	51.38			
Improved	5549	38.18			
Deteriorated	1517	10.44			

*N* = 14,534 young people from 75 services with 2–7,609 young people per service

^a^Age was recorded at the first available time point of routine data recording

^b^A descriptive comparison of referral source by ethnicity is shown in the supplementary material

^c^Case closure reason was recorded by the service

Each superordinate group can be delineated as follows. White British includes White British CYP only (67.61%,* n* = 9,826). White other includes Irish (0.63%, *n* = 91) and CYP from any other white background (4.70%, *n* = 683). Asian includes Indian (1.14%, *n* = 165), Pakistani (1.98%, *n* = 288), Bangladeshi (1.48%, *n* = 215), and CYP from any other-Asian background (1.46%, *n* = 212). Black includes Caribbean (3.85%, *n* = 560), African (4.14%, *n* = 602), and CYP from any other Black background (2.11%, *n* = 306). Mixed-race includes White and Black Caribbean (2.74%, *n* = 398), White and Black African (1.00%, *n* = 146), White and Asian (1.40%, *n* = 203), and CYP from any other mixed background (2.11%, *n* = 307). Other includes Chinese (1.04%, *n* = 151) and CYP from any other ethnic background (2.62%, *n* = 381).

### Measures

All quantitative variables were sourced from the merged CORC and CYP IAPT datasets. The five control independent variables (gender, age, referral source, presenting difficulty, and case closure reason) are clinical and demographic characteristics routinely collected by services.

The independent variable, ethnicity, was analysed in superordinate groups (Asian, Black, Mixed-race, Other, White other, and White British). All non-White British CYP were considered as being from minoritized ethnic backgrounds.

The data for the dependent variable, treatment outcome, are routinely collected by services using a multitude of standardised and idiographic outcome scales. Treatment outcome data were operationalised using the measurable change approach, an extension of the reliable change approach that allows for the inclusion of idiographic outcome scales, using the measures summarised in Table 2. Measurable change classifies individual-level changes in outcome measures from time 1 (T1) to time 2 (T2) by applying *reliable change* to standardised measures and considering change that is considered clinically important to allow for the inclusion of idiographic measures. Reliable change and change that is considered clinically important are outlined below.

**Table 2:** Outcome scales used by services

Outcome measure	Description
Strengths and Difficulties Questionnaire (SDQ) [56]	A short behavioural and emotional questionnaire for CYP. It consists of 25 items in 5 subscales to screen for: emotional problems, conduct problems, hyperactivity/inattention, peer relationships problems, and prosocial behaviour
Revised Child Anxiety and Depression Scale (RCADS) [57]	Monitor’s anxiety and depression across eight subscales (major depressive disorder, generalised anxiety disorder, obsessive–compulsive disorder, separation anxiety disorder, panic disorder, and social phobia). It has 47 items
Outcome Rating Scale (ORS) and the Child Outcome Rating Scale (CORS) [58]	Measures mental health and well-being holistically by assessing life functioning across four items: symptom distress, interpersonal well-being, social role, and overall well-being The CORS is a version of ORS for 6–12-year-olds
CORE-10 and Young Person CORE [59, 60]	These ten-item standardized tools are taken from the Clinical Outcomes in Routine Evaluation (CORE) set of outcome measures. The CORE-10 assesses anxiety, depression, trauma, functioning, physical problems, and risk to self. The Young Person CORE is a version for 11–16-year-olds
Generalised Anxiety Disorder assessment (GAD-7) [61]	Assesses Generalised Anxiety Disorder (GAD) severity across 7 items
Patient Health Questionnaire (PHQ-9) [62]	Monitor’s depression severity as treatment progresses for children and young people 13-year of age or older
The goal-based outcome tool (GBO) [40]	An *idiographic* goal-tracking measure. Up to 3 goals are rated in terms of progress from 0 (‘no progress towards goal’) to 10 (‘goal achieved’). It aims to enable goal agreement between therapist and patient and tracks progress towards them

Where a measure contained subscales, these were used for measurable change, as opposed to the measures total scores (i.e., SDQ, RCADS)

*CYP* children and young people

### Analytic strategy

Reliable change is a measure of the difference between an individual’s first-ever (T1), and last-ever (T2), score on a given treatment outcome scale, controlling for change attributable to that scale’s expected measurement error. To control for a scale’s measurement error, reliable change occurs only when a score passes a reliable change criterion calculated for each outcome scale: 1.96 × the Standard Error of the Difference (SE_diff_). SE_diff_, a variation of standard error, accounts for the two measurements made on the relevant outcome scale (at T1 and T2) and the internal consistency reliability of that relevant scale. The formula for SE_diff_ is displayed below, where *SD*_*time 1*_ is the standard deviation at T1 and *α* is the internal consistency reliability of the measure.

$${\mathrm{SE}}_{\mathrm{diff}}={SD}_{\mathrm{Time} 1} \times \sqrt{2}\times \sqrt{(1-\propto )}.$$

For standardised outcome measures CYP’s change from T1 to T2 was compared with that measure’s reliable change criterion. For idiographic outcome measures CYP’s change from T1 to T2 was compared with a *clinically important change* threshold defined by clinical experts [42] and used in previous analyses of the CORC dataset [63].

Individuals with scores exceeding the change criterion or clinically important change threshold on at least one scale, without measurably deteriorating on any other scale were deemed ‘measurably improved’. Individuals with scores reducing below the change criterion or clinically important change threshold on any measure were deemed ‘measurably deteriorated’. Individuals with scores showing no statistically reliable change or not passing the clinically important change threshold on any measure were deemed and as showing ‘no measurable change’ [39].

To investigate the association between ethnicity and CYP’s treatment outcomes (measurable change) controlling for gender, age, referral source, presenting difficulty, and case closure reason, multilevel multinomial logistic regressions were run in STATA 16 [64]. Multilevel modelling allowed for the control of service-level variation (e.g., CYP from the same service may be more similar than to those from different services).

Four preparatory models were run. The null model (Model 0) included treatment outcome only, to account for nesting of individuals within services. The intraclass correlation coefficient was 9% indicative of significant service-level variation and confirming that multilevel modelling was the appropriate statistical approach. Control variables were added in successive models with demographics, followed by clinical characteristics. For each categorical variable (excluding presenting difficulty), the reference category was the largest group. For each non-mutually exclusive presenting difficulty the reference category was the absence of that presenting difficulty, allowing for the inclusion of CYP with comorbidities.

Successive models were compared using likelihood ratio tests confirming each of the control variables should be retained in the final model. Adding gender and grand-mean centred age in Model 1(coded female and male; reference category: female) significantly improved the model fit, (*x*^2^ [4]) = 144.3, *p < *0.001. Adding referral source (reference category: primary care) and presenting difficulties in Model 2 significantly improved the model fit (*x*^2^[39]) = 205.67, *p < *0.001. Adding case closure reason (reference category: mutual agreement between patient and practitioner to end treatment) in Model 3 significantly improved the model fit (*x*^2^[8]) = 76.87, *p < *0.001. The final model added ethnicity (reference category: White British as it was the largest group).

### Qualitative data

#### Participants

In December 2020, 15 undergraduate students who had previous experience of mental health services were recruited via a university Participant Portal and were offered course credit for taking part. Participants were given an information sheet about the study and informed consent was obtained prior to the interviews. Overall, 13 participants were female, and 2 were male. The mean age was 19.19 (*SD* = 0.54); range 18–20 years. Most participants were from minoritized ethnic groups, with Asian (*n* = 8) and Indian groups (*n* = 3) being the most frequent. The remaining CYP were Mixed-race, White “other”, and White British. Recruitment ceased when the sample was sufficiently diverse.

#### Data collection

To be able to move beyond identifying ethnic differences in quantitative data, and understand what may be underpinning such differences, semi-structured interviews with a small number of young people from diverse ethnic backgrounds were conducted online via Microsoft Teams to help contextualise the findings based on their views and experiences. Interviews explored YP’s experiences of CYPMHS with questions such as “What were your experiences of ending CYPMHS support with a professional?”. To facilitate convergence between the data strands, the qualitative interviews transcripts were designed with the aim of understanding how participants’ experiences of ending support (qualitative) relate to treatment outcomes (quantitative). In particular, the aim was to explore experiences of young people from minoritized ethnic groups that may be associated with treatment outcomes. Accordingly, several questions tried to elucidate participants’ views on their outcomes: “Did you feel better or worse after ending treatment?”. To ensure data regarding thoughts and feelings were collected, each question was followed up by detailed questions and prompts (e.g., “why do you think this”) both predesignated and based on intuition.

### Analytic strategy

The interviews were audio recorded and transcribed verbatim, which formed the basis of the qualitative information for analysis. Thematic analysis [65, 66], was used to generate codes and then themes. All analysis was conducted by one researcher, with a second acting to verify the codes and themes.

## Results

### Quantitative results

Adding ethnicity to the final model significantly improved the model fit (*x*^2^[10]) = 22.73, p = 0.012. The final model output, in Table 3, has significant results in bold. Compared to White British CYP, CYP from Asian backgrounds (OR = 0.82, CI [0.70, 0.96]) and Mixed-race (odds ratio (OR) = 0.80; 95% CI [0.69, 0.92]) were less likely to measurably improve than not change. Effects of the control variables are discussed in the supplementary materials.

**Table 3:** Multilevel multinomial regression analysis with ethnicity predicting treatment outcome (effects of demographic and clinical characteristics shown in supplementary material). Significant results are in bold

Independent variable	Measurably improved vs. no change				Measurably deteriorated vs. no change			
	OR	*p* value	95% CI		OR	*p* value	95% CI	
			*LL*	*UL*			*LL*	*UL*
Ethnicity								
Asian vs. White British	**0.82**	0.015	0.70	0.96	1.07	0.534	0.86	1.35
Black vs. White British	0.95	0.477	0.84	1.08	1.13	0.173	0.95	1.36
Mixed-race vs. White British	**0.80**	0.003	0.70	0.93	0.98	0.880	0.79	1.22
Other vs. White British	0.88	0.221	0.73	1.08	0.82	0.246	0.60	1.14
White other vs. White British	0.90	0.237	0.77	1.07	1.07	0.549	0.84	1.38

Effects in bold are significant at the* p < *0.05 level*.* Effects of control variables available in supplementary material.* N* = 14,534 young people from 75 services with 2–7,609 young people per service

*OR* odds ratio, *CI* confidence interval, *LL* lower level, *UL* upper level

### Qualitative findings

The semi-structured interviews lasted approximately 20–30 min per participant. Three themes were generated, which consisted of (1) participants’ views about experiences that induce and constitute good endings and outcomes; (2) experiences of stigma that contribute to deterioration of participants’ condition, particularly for participants from minoritized ethnic backgrounds; and (3) perceived inequalities that induce premature treatment termination, particularly for participants from minoritized ethnic backgrounds. Participants typically described the latter two as leading to bad endings and outcomes.

## Discussion

This mixed methods study investigated whether CYP’s ethnicity was associated with treatment outcomes from CYPMHS. In terms of the quantitative findings, a multilevel multi-nominal regression analysis (controlling for age, gender, referral source, presenting difficulty, case closure reason) found that CYP from Mixed-race and Asian backgrounds were less likely to measurably improve than not change after treatment compared to White British CYP. This partially supports the hypothesis that CYP from minoritized ethnic backgrounds would have worse treatment outcomes than White British CYP. While some CYP from minoritized ethnic backgrounds (Asian and Mixed-race) were less likely to measurably improve compared to White British CYP, others did not (Black, Other, and White other). We held interviews with 15 young people from diverse ethnic backgrounds about their experiences of mental health support to help contextualise the findings of the quantitative analysis. Here, CYP described experiences of stigma and inequalities that may contribute to deterioration and poor outcomes, particularly for individuals from minoritized ethnic backgrounds. CYP viewed personalised support and the "right" therapist as conducive to good endings and valued outcomes pertaining to empowerment.

CYP value empowerment as a positive outcome. Indeed, previous literature identified empowerment and reduced symptoms as highly valued [49, 51]. The present qualitative findings further support this, where symptom changes were typically framed as a CYP’s ability, or empowerment, to cope with symptoms. Hence, empowerment appeared to be particularly valued. However, apart from one (*Youth Empowerment Scale-Mental Health),* most CYP mental health and well-being outcome measures focus on symptom changes rather than empowerment [67, 68]. This constrains existing measurement because failing to adequately conceptualise CYP’s valued outcomes may limit the ability of mental health support to meet their needs. Support may appear effective because an outcome measure demonstrates symptom improvement, but measurable change may not capture the nuanced outcomes, such as the importance CYP place on empowerment.

Participants also suggested that mismatches between practitioner and patient perceptions of desirable outcomes articulated as goals is unlikely to lead to positive changes. This supports the continued use of collaboratively framed goal-oriented practice, and goal-based outcome measures such as the GBO tool [69, 70] and the inclusion of idiographic measures in treatment outcome operationalisations. Notwithstanding, future research is needed to empirically investigate the appropriateness of combining different approaches to capturing outcome using both standardised and idiographic measures, as well as the exploration of measures for empowerment.

The quantitative finding that CYP from Mixed-race and Asian backgrounds were less likely to improve than not change compared to White British CYP contradicts previous findings that ethnicity has no association with treatment efficacy [34], and demonstrates that operationalising treatment outcome as an effect size may mask individual-level differences, supporting the continued use of individual-level operationalisations (i.e., meaningful change) [35]. This finding corroborates the proposal of an association between CYP’s ethnicity and outcomes (cf. introduction), based on previously observed structural inequalities in CYPMHS [8] and society [53]. Crucially, the findings did not suggest worse outcomes for all CYP from minoritized ethnic backgrounds. This may add confusion to the debate regarding whether individuals from Black backgrounds have worse outcomes than White British people, for instance on ending psychosis support [24, 71]. Pertinently, these findings raise questions about why individuals from Asian and Mixed-race backgrounds may experience worse treatment outcomes in the UK than CYP from other ethnic backgrounds. It should be noted that while the interviews we conducted help to contextualise the quantitative findings, they cannot provide definitive explanations, and rather raise potential areas for future exploration, such as this.

In the interviews, experiences constituting and facilitating good outcomes from mental health support for CYP from a range of ethnic backgrounds were explored. The extant literature suggests CYP across ethnicities value a perspective change and increased coping and resilience (likely facilitators of empowerment) [49]. Themes generated from discussions with CYP noted a progression to good outcomes initiated by a perspective change, increasing hope, improving coping and resilience, and ultimately inducing empowerment associated with symptom reductions. Crucially, if CYP from Asian and Mixed-race backgrounds were not guided through this progression, this may have contributed to their worse outcomes. Prioritising this process may be particularly helpful for redressing the worse outcomes experienced by CYP from Asian and Mixed-race ethnic backgrounds.

Experiences of stigma were discussed by participants as contributory to poor outcomes. Crucially, all CYP who discussed stigma in the interviews were from minoritized ethnic backgrounds, the majority being Asian and Mixed-race. This corroborates previous findings that Asian American and British Asian families are particularly prone to experiences of stigma, limiting accessing CYPMHS [4, 12, 15]. Stigma was described as CYP associating mental health conditions with shame and labels, which may lead to premature treatment termination, hence stigma may contribute to worse treatment outcomes experienced by Asian and Mixed-race CYP. This particular exposure to stigma experienced by CYP from some ethnic groups might explain the significant findings found for some groups (Asian and Mixed-race) and not for others (Black, Other and White other).

Second, wider inequalities may contribute to worse outcomes. Some CYP who were interviewed described decisions to end support was often a cost–benefit analysis between feeling better and financial constraints. Moreover, socioeconomic inequality was confounded by a perception that services had limited capacity, making support feel like a precious commodity. Consequently, some CYP ended support prematurely believing others needed it. Indeed, Asian CYP’s perception of services’ limited capacity exacerbated refraining from accessing care because of stigma. Hence, socioeconomic disadvantage may in part explain why Asian and Mixed-race CYP have worse outcomes than White British CYP. Socioeconomic inequalities represent a small manifestation of wider societal inequalities that disadvantage CYP from minoritized ethnic backgrounds, including contexts outside of the UK. Racial and ethnic discrimination has severe consequences for the health of minoritized ethnic groups [72]. This may be overt, or covert where racism is embedded in society (systemic racism) [73]. Crucially, health-care occurs in a society with embedded ethnic inequalities [74]. As it has been suggested that systemic racism influences how CYP from minoritized ethnic backgrounds access care [23], such inequalities likely also influence outcomes and experiences of ending treatment. This is an urgent area for future research, especially to provide evidence-based recommendations for services to go beyond culturally competent care, to providing care that recognises the influence of wider ethnic inequalities on individuals from minoritized ethnic groups [53].

While the main strength of this research is its inclusion of CYP from diverse minoritized ethnic backgrounds, there are also limitations. First, past research suggests that socioeconomic deprivation may explain some differentiation in experiences of CYP from different ethnic backgrounds [8]. Not controlling for socioeconomic status in the present research is limiting and should be considered in future research. Secondly, due to the paucity of similar research, it is difficult to consider the application of the findings to a non-UK context, as the study included data from UK CYPMHS and may not generalise to non-UK services. The underrepresentation of males in the qualitative sample means that there might be specific experiences relating to males and intersectionality that it has not been possible to explore. However, as with all qualitative research, the aim is not to generalise these findings but to learn from the experiences of the participants. Finally, people from minoritized ethnic backgrounds have been historically underrepresented in services and research [75]. Consequently, the study may have struggled to appropriately represent voices of most interest.

In conclusion, this research suggests that CYP from Asian and Mixed-race minoritized ethnic backgrounds have worse treatment outcomes from CYPMHS compared to White British CYP. Future quantitative research replicating this study, should also potentially explore prevalence rates i.e., mental health conditions, as a study variable; assessing whether ethnicity may have a differential effect on treatment outcomes depending on the condition. Any qualitative research should elucidate experiences contributing to the worse outcomes experienced by Asian and Mixed-race CYP, and any differences across CYP from other minoritized ethnic backgrounds. Importantly, the available relevant research in the surrounding literature is based in US/UK settings, which is a limitation in this area’s knowledge. The inclusion of idiographic measures was deemed particularly beneficial. Meanwhile, the lack of measures including empowerment should be a focus of future attention. Future practice should include discussions about patients’ desired outcomes at therapy initiation to ensure these are matched appropriately. This may improve outcomes for all and potentially help redress the less favourable outcomes experienced by Asian and Mixed-race CYP.

### Supplementary Information

Below is the link to the electronic supplementary material.

Supplementary file1 (DOCX 29 KB)

## Data Availability

The administrative data used in the present research can be made available upon request. Please contact the CORC team at corc@annafreud.org.
