# Supplementary material for: Ethnic differences in treatment outcome for children and young people accessing mental health support
Source: Eur Child Adolesc Psychiatry. 2023 May 28;33(4):1121–31. doi: 10.1007/s00787-023-02233-5 (PMC11032270; doi:10.1007/s00787-023-02233-5)
Supplement: Supplementary file 1 — Supplementary file1 (DOCX 29 KB) [file 787_2023_2233_MOESM1_ESM.docx]

**Ethnic differences in treatment outcome for children and young people accessing mental health support:**

**Supplementary material**

**Study findings for control variables**

The results of the multilevel multinomial regression analysis with demographic and clinical characteristics predicting treatment outcome are shown in Table S1. While gender was not, age was a significant predictor of treatment outcome. Compared to younger CYP, older CYP were less likely to measurably improve or deteriorate than not change.

In terms of referral source, compared to CYP referred through primary care, CYP who self-referred were less likely to measurably deteriorate than not change. Compared to CYP referred through primary care, CYP referred through education, mental health, or with no reported referral source were more likely to measurably deteriorate than not change.

In terms of presenting difficulties, CYP with learning disability or ‘other’ problems were more likely to measurably improve than not change compared to CYP without these presenting difficulties. CYP with conduct problems were less likely to measurably improve than CYP without conduct problems. CYP with emotional problems were more likely to measurably deteriorate than not change compared to CYP without emotional problems. CYP with ‘other’ problems were less likely to measurably deteriorate than not change compared to CYP without ‘other’ problems.

In terms of case closure reason, compared to CYP who mutually agreed with their practitioner to end support (mutual agreement), CYP whose case closure reason was nonattendance were less likely to measurably improve and more likely to measurably deteriorate than not change. Compared to CYP who mutually agreed to end support, CYP whose case closure reason was onward referral were less likely to measurably improve and more likely to measurably deteriorate than not change. Compared to CYP who mutually agreed to end support, CYP who ended support for an ‘other case closure reason’ were less likely to measurably improve than not change. Compared to CYP who mutually agreed to end support, CYP with a missing case closure reason were less likely to measurably improve and more likely to measurably deteriorate than not change.

Table S1. Multilevel multinomial regression analysis with demographic and clinical characteristics predicting treatment outcome. Significant results are in bold.

| Independent variable | Measurably improved vs. no change | | | | | | | Measurably deteriorated vs. no change | | | | | | | |
| --- | --- | --- | --- | --- | --- | --- | --- | --- | --- | --- | --- | --- | --- | --- | --- |
|  | OR | p-value | | | 95% CI | | | OR | | p-value | | | | 95% CI | |
|  |  |  | | | LL | | UL |  | |  | | | | LL | UL |
| **Demographics** |  | |  | |  | |  |  | | | |  | |  |  |
| Female vs. male | 1.02 | 0.54800 | | | 0.95 | | 1.10 | 0.93 | | 0.22500 | | | | 0.83 | 1.05 |
| Age | **0.94** | 0.00000 | | | 0.92 | | 0.95 | **0.90** | | 0.00000 | | | | 0.88 | 0.93 |
| **Referral source** |  |  | |  | |  | | |  | |  | |  |  |  |
| Self vs. primary care | 0.92 | 0.38600 | | | 0.77 | | 1.10 | **0.69** | | 0.00800 | | | | 0.52 | 0.91 |
| Education vs primary care | 1.10 | 0.29300 | | | 0.92 | | 1.32 | **1.39** | | 0.01800 | | | | 1.06 | 1.82 |
| Social care/ youth justice | 1.03 | 0.81600 | | | 0.78 | | 1.37 | 1.46 | | 0.06900 | | | | 0.97 | 2.21 |
| Child health vs. primary care | 1.09 | 0.51300 | | | 0.84 | | 1.43 | 0.93 | | 0.76000 | | | | 0.57 | 1.50 |
| Mental health vs. primary care | 1.04 | 0.64000 | | | 0.87 | | 1.25 | **1.38** | | 0.02600 | | | | 1.04 | 1.84 |
| Other vs. primary care | 0.97 | 0.81500 | | | 0.73 | | 1.29 | 1.01 | | 0.66000 | | | | 0.70 | 1.75 |
| Not reported vs. primary care | 1.07 | 0.45400 | | | 0.90 | | 1.25 | **1.37** | | 0.00700 | | | | 1.09 | 1.73 |
| **Presenting difficulties** |  |  | |  | |  | | |  | |  | |  |  |  |
| Child in need | 1.07 | 0.59100 | | | 0.84 | | 1.35 | 1.08 | | 0.67600 | | | | 0.74 | 1.58 |
| Child protection plan | 1.02 | 0.78500 | | | 0.87 | | 1.20 | 1.19 | | 0.13500 | | | | 0.95 | 1.50 |
| Autism | 0.93 | 0.57000 | | | 0.71 | | 1.21 | 0.75 | | 0.24500 | | | | 0.46 | 1.22 |
| Conduct problems | **0.78** | 0.00100 | | | 0.67 | | 0.91 | 0.84 | | 0.17100 | | | | 0.66 | 1.08 |
| Developmental difficulties | 1.00 | 0.98000 | | | 0.75 | | 1.32 | 1.04 | | 0.85700 | | | | 0.65 | 1.67 |
| Eating disorder | 1.09 | 0.28200 | | | 0.93 | | 1.28 | 1.20 | | 0.15200 | | | | 0.93 | 1.55 |
| Emotional problems | 1.11 | 0.10300 | | | 0.98 | | 1.25 | **1.57** | | 0.00000 | | | | 1.30 | 1.89 |
| Repetitive behaviour problems | 1.12 | 0.12100 | | | 0.97 | | 1.29 | 0.90 | | 0.36300 | | | | 0.71 | 1.14 |
| Hyperactivity | 1.12 | 0.40800 | | | 0.85 | | 1.48 | 0.70 | | 0.14200 | | | | 0.43 | 1.13 |
| Learning difficulties | **1.46** | 0.00500 | | | 1.12 | | 1.90 | 1.02 | | 0.90700 | | | | 0.64 | 1.65 |
| Psychosis | 0.87 | 0.17900 | | | 0.72 | | 1.06 | 1.24 | | 0.17100 | | | | 0.91 | 1.67 |
| Self-harm | 1.05 | 0.48100 | | | 0.92 | | 1.19 | 1.03 | | 0.77700 | | | | 0.84 | 1.27 |
| Substance use | 1.16 | 0.28600 | | | 0.88 | | 1.53 | 1.28 | | 0.24000 | | | | 0.85 | 1.94 |
| Other problems | **1.19** | 0.00400 | | | 1.06 | | 1.34 | **0.78** | | 0.00700 | | | | 0.65 | 0.93 |
| **Case closure reason** |  |  | |  | |  | | |  | |  | |  |  |  |
| Nonattendance vs. mutual agreement | **0.58** | 0.00000 | | | 0.47 | | 0.72 | **1.36** | | 0.03700 | | | | 1.02 | 1.81 |
| Onward referral vs. mutual agreement | **0.68** | 0.00300 | | | 0.52 | | 0.88 | **1.43** | | 0.06200 | | | | 0.98 | 2.07 |
| Other case closure reason vs. mutual agreement | **0.66** | 0.00100 | | | 0.52 | | 0.83 | 0.73 | | 0.15100 | | | | 0.48 | 1.12 |
| Missing case closure reason vs. mutual agreement | **0.83** | 0.00900 | | | 0.72 | | 0.95 | **1.34** | | 0.00500 | | | | 1.09 | 1.65 |
| **Ethnicity** |  |  | | |  | |  |  | |  | | | |  |  |
| Asian vs. White British | **0.82** | 0.01400 | | | 0.70 | | 0.96 | 1.08 | | 0.53300 | | | | 0.86 | 1.35 |
| Black vs. White British | 0.95 | 0.43900 | | | 0.84 | | 1.08 | 1.14 | | 0.14900 | | | | 0.95 | 1.37 |
| Mixed-race vs. White British | **0.80** | 0.00200 | | | 0.69 | | 0.92 | 0.10 | | 0.99200 | | | | 0.81 | 1.24 |
| Other vs. White British | 0.87 | 0.15900 | | | 0.71 | | 1.06 | 0.81 | | 0.20900 | | | | 0.59 | 1.12 |
| White-other vs. White British | 0.89 | 0.18200 | | | 0.76 | | 1.05 | 1.09 | | 0.49500 | | | | 0.85 | 1.39 |

*Note. N =* 14,534 young people from 75 services with 2-7,609 young people per service. OR = odds ratio. CI = confidence interval. LL = lower level. UL = upper level. Effects in bold are significant at least at the *p* < 0.05 level.

Table S2. Frequency and row percentages of referral source by ethnicity.

| Ethnicity |  | Primary care | Self-referral | Education | Social care/ youth justice | Child health | Mental health | Other | Not reported | Total |
| --- | --- | --- | --- | --- | --- | --- | --- | --- | --- | --- |
| Asian | *n* | 125 | 109 | 99 | 25 | 11 | 43 | 18 | 450 | 880 |
|  | Row % | 14.2 | 12.39 | 11.25 | 2.84 | 1.25 | 4.89 | 2.05 | 51.14 | 100 |
| Black | *n* | 92 | 237 | 125 | 49 | 24 | 38 | 18 | 885 | 1,468 |
|  | Row % | 6.27 | 16.14 | 8.51 | 3.34 | 1.63 | 2.59 | 1.23 | 60.29 | 100 |
| Mixed-race | *n* | 128 | 214 | 94 | 27 | 11 | 37 | 15 | 528 | 1,054 |
|  | Row % | 12.14 | 20.3 | 8.92 | 2.56 | 1.04 | 3.51 | 1.42 | 50.09 | 100 |
| Other | *n* | 94 | 109 | 49 | 14 | 11 | 30 | 12 | 213 | 532 |
|  | Row % | 17.67 | 20.49 | 9.21 | 2.63 | 2.07 | 5.64 | 2.26 | 40.04 | 100 |
| White-other | *n* | 134 | 128 | 83 | 26 | 14 | 42 | 12 | 335 | 774 |
|  | Row % | 17.31 | 16.54 | 10.72 | 3.36 | 1.81 | 5.43 | 1.55 | 43.28 | 100 |
| White British | *n* | 2,065 | 1,348 | 710 | 171 | 233 | 682 | 189 | 4,428 | 9,826 |
|  | Row % | 21.02 | 13.72 | 7.23 | 1.74 | 2.37 | 6.94 | 1.92 | 45.06 | 100 |
| Total | *n* | 2,638 | 2,145 | 1,160 | 312 | 304 | 872 | 264 | 6,839 | 14,534 |
|  | Row % | 18.15 | 14.76 | 7.98 | 2.15 | 2.09 | 6 | 1.82 | 47.06 | 100 |

*Note. N =* 14,534 young people from 75 services with 2-7,609 young people per service.
